# The FGF/FGFR/c-Myc axis as a promising therapeutic target in multiple myeloma

**DOI:** 10.1186/s13046-024-03217-2

**Published:** 2024-11-01

**Authors:** Arianna Giacomini, Sara Taranto, Giorgia Gazzaroli, Jessica Faletti, Davide Capoferri, Raffaella Marcheselli, Margherita Sciumè, Marco Presta, Antonio Sacco, Aldo M. Roccaro

**Affiliations:** 1 Department of Molecular and Translational Medicine, University of Brescia, Brescia, Italy; 2 Clinical Trial Center, Translational Research and Phase I Unit, ASST Spedali Civili Di Brescia, Brescia, Italy

**Keywords:** Multiple myeloma, C-Myc, FGF/FGFR system

## Abstract

Among blood cancers, multiple myeloma (MM) represents the second most common neoplasm and is characterized by the accumulation and proliferation of monoclonal plasma cells within the bone marrow. Despite the last few decades being characterized by the development of different therapeutic strategies against MM, at present such disease is still considered incurable. Although MM is highly heterogeneous in terms of genetic and molecular subtypes, about 67% of MM cases are associated with abnormal activity of the transcription factor c-Myc, which has so far revealed a protein extremely difficult to target. We have recently demonstrated that activation of fibroblast growth factor (FGF) signaling protects MM cells from oxidative stress-induced apoptosis by stabilizing the oncoprotein c-Myc. Accordingly, secretion of FGF ligands and autocrine activation of FGF receptors (FGFR) is observed in MM cells and FGFR3 genomic alterations represent some 15–20% MM cases and are associated with poor outcome. Thus, FGF/FGFR blockade may represent a promising strategy to indirectly target c-Myc in MM.

On this basis, the present review aims at providing an overview of recently explored connections between the FGF/FGFR system and c-Myc oncoprotein, sustaining the therapeutic potential of targeting the FGF/FGFR/c-Myc axis in MM by using inhibitors targeting FGF ligands or FGF receptors. Importantly, the provided findings may represent the rationale for using FDA approved FGFR TK inhibitors (i.e. Pemigatinib, Futibatinib, Erdafitinib) for the treatment of MM patients presenting with an aberrant activation of this axis.

## Introduction

Multiple myeloma (MM) is a rare tumor characterized by the uncontrolled proliferation of mature monoclonal plasma cells within the bone marrow (BM). It is the second most common blood cancer in Europe and in the United States [1], with an estimate of about 35,730 newly diagnosed MM cases in the United States in 2023 (source: www.cancer.org).

MM usually occurs in the third-age population and is more frequent in men than women [1, 2]. Over time, the growing number of studies and research on MM has certainly provided novel insights for improving the management of this disease [3]. However, despite the continuous improvements, MM remains incurable, and most of the patients succumb due to disease progression.

Active MM is an advanced stage of cancer preceded by two known asymptomatic stages, the former named MGUS (monoclonal gammopathy of undetermined significance) and the latter SMM (smoldering multiple myeloma) [4, 5]. MGUS is characterized by an abnormal accumulation of serum monoclonal component (MC) in the peripheral blood and it is considered as a premalignant precursor stage of MM [4]. Patients with MGUS gradually progress towards MM, with a risk of 1% per year. On the other hand, patients affected by SMM show elevated levels of serum MC together with an enrichment of plasma cells within the BM (over 60%) [5]. SMM is also a premalignant condition, associated with a higher risk of progression towards MM compared with MGUS (10% per year). SMM is believed to be an intermediate phase between MGUS and MM [6].

Several genomic events drive the development and progression of MM, thus confirming the occurrence of complex and heterogeneous multistep transformation processes supporting MM pathogenesis. Indeed, the acquisition of genomic alterations supports the evolution of polyclonal plasma cells (typic of physiologic conditions) in monoclonal plasma cells, characterizing the MGUS stage. Primary oncogenic events occur in the germinal center of B cells and are associated with the establishment of the MGUS clone. The main primary events are IGH translocations and trisomy [7, 8]. From a cytogenetic point of view, MM is usually classified as hyperdiploid or non-hyperdiploid [9]. The hyperdiploidy karyotype may involve chromosomes 3, 5, 7, 9, 11, 15, 19 and 21, resulting in gene overexpression and, consequently, in abnormal growth and replication of cells [10]. The hyperdiploidy accounts for 50–60% of cases and is associated with poor outcomes [9]. On the other hand, the non-hyperdiploid subset presents translocation between the locus of immunoglobulin heavy chain (IgH) on chromosome 14q32 and one of the several MM-related oncogenes. Such translocation frequently involves the MAF bZIP transcription factor (MAF), the fibroblast growth factor receptor 3 (FGFR3), the multiple myeloma SET domain (MMSET), and cyclin D1 and D3, leading to the upregulation of cyclin D protein [11].

These mechanisms are necessary but not sufficient for the transition from MGUS/SMM to active and symptomatic MM [5]. After the setting of MGUS stage, secondary events are required for the initiation of MM, including further translocations, mutations, deletions and others [12]. The consequences are the activation of oncogenes (e.g., RAS mutations) [13] and/or inactivation of oncosuppressor genes (e.g., deletion of TP53) [14, 15]. Among the plethora of secondary oncogenic events, aberrations of *MYC* gene on chromosome 8q24 and its overexpression seem to play a pivotal role in the pathogenesis of MM [16, 17]. Indeed, deregulated *MYC* expression represents one of the key features of disease progression and the overexpression of the oncoprotein c-Myc is associated with poor prognosis and inferior overall survival of MM patients [16, 18]. In this context, we have recently demonstrated that activation of fibroblast growth factor (FGF) signaling is involved in the stabilization of c-Myc oncoprotein in MM cells, strongly supporting the existence of an FGF/FGFR/c-Myc axis that may play a pivotal role in the progression of this hematologic disease [19].

The purpose of this review is to to provide an overview of recently explored connections between the FGF/FGFR system and c-Myc oncoprotein, thus sustaining the therapeutic potential of targeting the FGF/FGFR/c-Myc axis in MM.

## The proto-oncogene *MYC*

*MYC* gene is located on chromosomes 8q24 and is composed by 3 exons that codify for several *MYC* transcripts (splice variants). Among these transcripts only two (3721 bp and 2150 bp) give rise to the production of two functional isoforms of the c-Myc protein [20]. c-Myc is a transcription factor involved in the regulation of about 10 –15% of all human genes leading to the control of a multitude of cellular functions, including cell cycle progression, cell growth, cell survival, self-renewal, cellular metabolism and biosynthesis, ribosome biogenesis, cell adhesion, mitochondrial function, as well as extracellular processes affecting the local microenvironment [21].

### Regulation of *MYC* gene expression and c-Myc protein activity

Because of its pivotal role in several cell functions, *MYC* gene expression and c-Myc protein activity are tightly regulated at different levels. First, the regulating mechanisms that control *MYC* transcription are extremely complex. Indeed, multiple promoters (P0, P1, P2 and P3) participate in *MYC* transcription but primary it predominantly initiates from two major promoters, P1 and P2. Beyond that, *MYC* promoters are regulated by two noncanonical cis-regulatory elements, FUSE and CT element which respond to torsional stress caused by transcription activation and facilitate the formation of alternate DNA structures, respectively [22]. Also, several transcription factors involved in different pathways (e.g., SP1, NFAT, CNBP, p53) and transcriptional co-activators (e.g., DDX5 and BRD4) can influence *MYC* transcription by interacting with different regions of *MYC* gene [22, 23]. After transcription, multiple factors including miRNAs (e.g., miRNA let-7 and miR-145), RNA binding proteins (e.g., FXR1 and IGF2BP) [22] and long noncoding RNAs (e.g., GHET1, LINC-ROR, lncRNA-MIF and lncRNA GAS5), control post-transcriptional regulation of *MYC* mRNA [24].

**Fig. 1:**
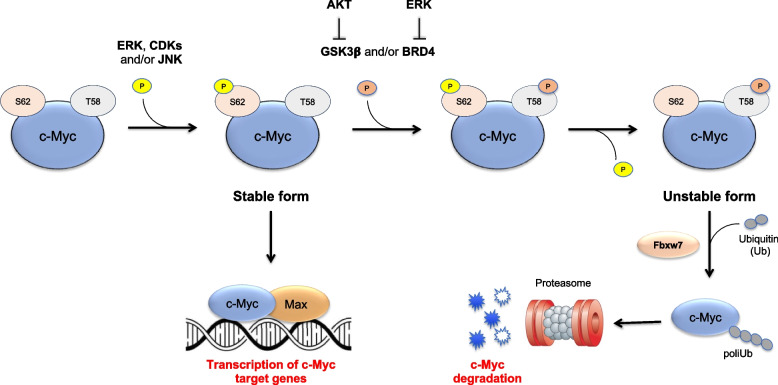
Mechanisms of c-Myc protein stabilization and degradation. The phosphorylation of c-Myc at Serine 62 (S62) residue determines protein stabilization, c-Myc translocation into the nucleus and increased c-Myc transcriptional activity. S62 phosphorylation is essential for the subsequent phosphorylation at threonine 58 (T58) residue which in turns induces the removal of the phosphate group at S62. The singly phosphorylated T58-Myc is unstable being finally recognized by the Fbxw7 ubiquitin ligase and degraded by the 26S proteasome. S62 is phosphorylated by different types of kinases, including ERK, CDKs, and JNK, whereas T58 can be phosphorylated by GSK-3β kinase or by BRD4 protein [30]. T58 phosphorylation by GSK-3β and BRD4 can be inhibited by AKT and ERK, respectively

Finally, c-Myc can be regulated at protein level by post-translational modifications influencing c-Myc stability. Indeed, c-Myc protein has a short half-life of ∼30 min in proliferating cells being continuously subjected to cycles of phosphorylation and dephosphorylation that regulate its degradation through the ubiquitin–proteasome pathway [25]. There are two different conserved phosphorylation sites within the Myc Box (MB) I region of c-Myc protein, threonine 58 (T58) and serine 62 (S62), which are part of a phospho-degron motif recognized by Fbw7, the E3 ubiquitin ligase deputized to c-Myc proteasomal degradation [26]. The phosphorylation of c-Myc at S62 residue determines protein stabilization, c-Myc translocation into the nucleus and increased c-Myc transcriptional activity. Indeed, c-Myc phosphorylated at S62 associates with the protein partner MAX to form a heterodimer, which, in turns, binds to the E-box elements together with other co-activators and enhancers, driving the transcription of proliferative and anti-apoptotic target genes. In addition, S62 phosphorylation is essential for the subsequent c-Myc phosphorylation at T58 residue which in turns induces the removal of the phosphate group at S62 [27]. This is an essential step to induce c-Myc degradation. Indeed, the unstable singly phosphorylated T58-Myc can be recognized by the Fbxw7 ubiquitin ligase and degraded by the 26S proteasome [28] (Fig. 1). Based on this phosphorylation and dephosphorylation mechanism, c-Myc protein levels can be influenced by several signaling pathways which target the modification of S62 and T58 sites. Indeed, S62 is phosphorylated by different types of kinases, including ERK, CDKs, and JNK [29], whereas T58 can be phosphorylated by GSK-3β [27] or by BRD4 [30] kinases. Importantly, T58 phosphorylation by GSK-3β and BRD4 can be inhibited by AKT and ERK, respectively (Fig. 1) [25, 31].

### Deregulation of *MYC* gene expression and c-Myc protein activity in cancer

Deregulation of *MYC* gene expression and c-Myc protein levels have been associated with numerous diseases including cancer. Indeed, *MYC* is one of the first oncogene identified in human cancer, described as a cellular homolog of the avian retroviral oncogene v-myc. Among the other members of its family, *MYCN* and *MYCL*, *MYC* is the most expressed and deregulated in cancers [20]. It is now well known that deregulated c-Myc activity promotes tumor progression enforcing many of the hallmark features of cancer, including uninterrupted tumor cell proliferation and growth, protein synthesis, and altered cellular metabolism. Also, the oncoprotein c-Myc is involved in stemness induction, cellular senescence and differentiation blockade, tumor metastasis and tumor cell resistance to chemotherapy [32, 33]. Additionally, recent studies have shown that c-Myc is also a crucial regulator of tumor microenvironment orchestrating changes like activation of angiogenesis and suppression of the host immune response [32, 34].

The oncogenic activation of c-Myc can occur at different levels. First, transformation of normal cells into tumor cells can occur via *MYC* gene overexpression. This can be caused by two main factors, genomic aberrations (direct mechanism) or upregulation of *MYC* gene expression by cellular pathways (indirect mechanism). Regarding the former, three major genomic aberrations are responsible for *MYC* overexpression: insertional mutagenesis, gene amplification, and chromosomal translocation. Insertional mutagenesis occurs in retrovirus-induced tumors. For example, the proviral enhancer of the avian leucosis virus (ALV), which induces hematopoietic tumors, can be integrated upstream of the *MYC* gene leading to c-Myc overexpression [35]. As concerns *MYC* gene amplifications, they are found in both hematopoietic and non-hematopoietic tumors [36]. At last, chromosomal translocations involving *MYC* usually juxtaposes its gene locus (8q24) to immunoglobulin genes at chromosome 14q32, 2p11, and 22q11 or other partner genes [37]. As to *MYC* upregulation by activation of intracellular pathways, increased *MYC* gene expression can be the consequence of the activation of other oncogenes, including *RAS*, *SRC*, *NOTCH* or inactivation of tumor suppressor genes such as *APC* [38]. Interestingly, several studies revealed that distinct *MYC* expression thresholds determine the effect of c-Myc in oncogenesis. For example, dysregulated expression at low levels may cause limited ectopic proliferation. Slightly higher levels of *MYC* expression could activate the ARF/TP53 axis and tumor suppressor pathways that limit the effect of c-Myc in the absence of other oncogenic events. Finally, highly dysregulated overexpression of *MYC* is able to induce the formation of sporadic tumors in animal models as a consequence of *TP53* and/or apoptotic inactivation mechanisms [39].

Beyond overexpression, point mutations in *MYC* gene coding sequence can influence the post-translational regulation mechanisms causing an increase of c-Myc protein stability. These type of mutation in *MYC* gene are rarely found in solid tumors, but more frequently in lymphomas, such as human Burkitt's lymphoma, AIDS-associated lymphomas, and certain acute lymphoblastic leukemias [40]. Hot spots for these mutations are found within or near the evolutionary conserved Myc box 1, the residue most frequently mutated being T58. Mutations at T58 lead to the synthesis of stable mutant c-Myc protein that cannot be degraded and thus with increased transforming potential [41].

Finally, in absence of mutations in the codons codifying for S62 and T58, increased c-Myc oncoprotein level can be due to post-translational modifications in the phosphorylation status of these two aminoacidic residues upon activation of oncogenic pathways. For instance, the hyperactivation of MAPK and Akt signaling, frequently observed in cancer, is responsible for increased phosphorylation at S62 and decreased phosphorylation at T58, thus influencing c-Myc stability, degradation and half-life [42, 43]. In this context, it has been recently reported by us that the Fibroblast Growth Factor (FGF) signaling may exert a pivotal role in regulating the stability of c-Myc oncoprotein, as discussed in the next paragraphs [19, 42, 44].

## Impact of *MYC* deregulation in MM progression

**Fig. 2:**
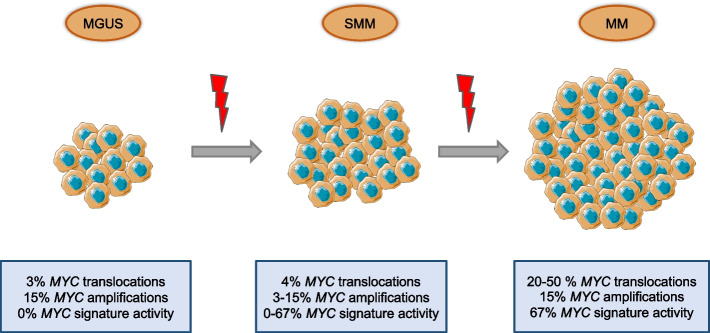
The oncogene MYC activation during MGUS to MM progression. Reported are the percentage of cases presenting genomic alteration of *MYC* gene (chromosomal translocation / gene amplification) or altered activation of *MYC* signature

As already mentioned, MM evolves from the premalignant asymptomatic stage MGUS, followed by SMM, to active multiple myeloma [45]. The mutational landscape of the events driving MM development is wide, but interestingly data from genomic and gene set enrichment analyses indicated that the oncogene *MYC* and its signature are mostly deregulated in active MM cases than in MGUS and SMM [46] (Fig. 2), suggesting that *MYC* deregulation may be one of the key events triggering the transition between MGUS to full-blown MM [47, 48].

The pivotal role of c-Myc in the MGUS to MM progression was first established through a *MYC*-driven MM transgenic mouse model, named Vk*MYC, where the transgene *MYC* was put under the control of the regulatory elements of the kappa light chain (Vk) gene. In this model, the third codon of Vk was mutated in order to generate a stop codon creating a DGYW motif, which is the target sequence for somatic hypermutation. Therefore, the sporadic *MYC* activation could occur only in B cells undergoing Activation-induced cytidine deaminase (AID)-dependent somatic hypermutation (SHM) leading to reverted stop codon. This model was able to fully recapitulate the clinical and pathological features of MM disease, demonstrating that c-Myc can drive the onset of MM. Indeed, *MYC* activation induced the progression from MGUS to MM in an in vivo MGUS model that rarely develops MM, confirming its role as an oncogenic driver event [49].

The role of *MYC* in MM early-stage disease progression was confirmed also by other studies [46]. For instance, the increasing *MYC* deregulation during disease stage progression (Fig. 2) seems to go along with the rate of cell proliferation. Indeed, MGUS samples present less than 1% of Ki67 positive plasma cells, while newly diagnosed MM tumors present 2% to 10% Ki67 positive tumor cells, suggesting that c-Myc expression may be important in affecting cell proliferation and energy metabolism in rapidly proliferating plasma cells [50]. In this context it is worthy to mention that c-Myc hyperactivation increases replication stress and genomic instability promoting cancer progression and drug resistance [51, 52]. Indeed, to make cancer cell survive the DNA damage overload due to oncogenic stress, c-Myc protects cancer-cell genome by promoting the transcription of several genes involved in double-strand breaks repair, including poly (ADP-ribose) polymerase (PARP) [53]. Interestingly, Caracciolo et al. have recently demonstrated the addiction of MYC-driven MM cells to PARP1, being high *MYC* expression positively correlated with sensitivity to PARP inhibitors [54].

Based on these findings, several studies have been conducted to get deeper insights into *MYC* deregulation mechanisms in MM cells and how this affects MM progression. Now it is known that *MYC* deregulation occurs at different levels in MM cells leading to *MYC* overexpression, increased c-Myc translation and c-Myc protein stability.

### Mechanisms involved in *MYC* overexpression in MM

Clinical and biological data have shown that *MYC* overexpression can be the consequence of *MYC* locus rearrangements and amplifications in MM. The rearrangements (including insertions, inversions, and translocations) of *MYC* gene are detected by FISH and are associated with high disease burden (elevated β2 microglobulin in MM patients’ blood, ISS stage II/III, extramedullary disease, and presence of a plasmablastic morphology) [17]. Especially, *MYC* translocations have been identified in 20 – 50% of patients with newly diagnosed MM [55], while they were rarely detected in patients with MGUS and SMM (3% and 4%, respectively) [56, 57] (Fig. 2). *MYC* translocations involve preferentially the immunoglobulin (IG) loci (IGH > IGL > IGK), but also non-Ig partners (including FAM46C, FOXO3, and BMP6) leading to the transcriptional control of *MYC* gene by enhancers or super-enhancers and finally resulting in overexpression of the oncogene [58]. Also, clinical data revealed that MM patients bearing *MYC* translocations have decreased progression-free survival (PFS) and overall survival (OS) independently of age, advanced stage, or cooccurrence of high-risk cytogenetic abnormalities [58]. On the other hand, *MYC* amplifications at 8q24.21 locus are present from MGUS to full-blown myeloma in about 15% of patients and associate with poor prognosis [59] (Fig. 2).

### Mechanisms regulating *MYC* transcription in MM

Several studies have established that *MYC* overexpression can also occur after an increment of *MYC* transcription mediated by different factors such as BET bromodomain proteins, bone morphogenetic proteins (BMPs) and the interferon regulatory factor 4 (IRF4). It has been shown that BET bromodomain proteins may act as key players in the regulation of *MYC* expression. Indeed, JQ1, a selective small-molecule bromodomain inhibitor, was able to inhibit both *MYC* transcription and *MYC*-related target genes, thus leading to inhibition of MM cell proliferation both in vitro and in vivo. Specifically, the JQ1-dependent anti-MM activity resulted from induction of cellular senescence and cell cycle arrest [60]. BMPs also seem to regulate *MYC* expression. BMPs can potently induce MM cell death and a study by Holien et al. showed that BMP-induced apoptosis is correlated with lowered *MYC* mRNA and protein levels. Indeed, they demonstrated that BMPs can induce MM cells apoptosis through c-Myc downregulation by the Smad pathway [61, 62].

Another important factor that leads to activation of *MYC* gene is IRF4, a transcription factor involved in plasma cell differentiation and class switch recombination belonging to the interferon regulatory factor (IRF) family [63]. Louis M. Staudt et al. recently revealed that *IRF4*-mediated *MYC* transcription is essential also in MM. Indeed, *IRF4*-silencing by RNA interference-based genetic screen has been shown to enhance MM cell apoptosis and to decrease *MYC* mRNA. In addition, transcriptome profiling and genome-wide chromatin immunoprecipitation analysis of IRF4-binding sites revealed that *MYC* is one of the direct target gene of IRF4 in MM cells. It was also showed that IRF4 and c-Myc form a positive autoregulatory loop in MM, since c-Myc can transactivate IRF4. Accordingly, myeloma patient samples were shown to present a significantly higher expression of both *MYC* and *IRF4* mRNA as compared to the normal plasma cells, thus further suggesting the existence of a positive correlation between *IRF4* and *MYC* expression levels in MM [64]. In this context, immunomodulatory drugs (IMiDs) have been shown to inhibit both *MYC* and *IRF4* transcription in MM cells by activating the Ikaros axis [65]. Indeed, IMiDs induce the proteasome degradation of the transcription factors Ikaros (IKZF1) and Aiolos (IKZF3), thus downregulating the expression of IKZF1/3 target genes, including *MYC* and *IRF4*. In addition, other transcription factors like ETV4 [66] and BATF2 [67] have been recently identified to induce *MYC* expression independently of Ikaros axis, thus contributing to IMiD resistance in MM.

### Mechanisms regulating *MYC* translation in MM

Beyond *MYC* transcription regulation, also *MYC* mRNA regulation by miRNAs (such as Let-7 and miR-22) and RNA binding proteins (such as 4EBP1) can be responsible for c-Myc upregulation in MM cells. For instance, different miRNAs and specifically the Let-7 miRNAs family are involved in the regulation of *MYC* expression. In particular, the LIN28B/Let-7/MYC axis has been reported in several types of cancers, including MM. LIN28B is the RNA-binding protein that impairs the processing of Let-7 precursors into mature, functional miRNAs. In physiological conditions, Let-7 miRNAs function as tumor suppressor through transcriptional repression of key oncogenes, including *MYC* and *RAS*, by binding specific sites in the 5′-untranslated region (UTR) and activating the RNA-induced silencing complex [68]. However, in MM a deregulation of LIN28B has been observed that, in turns, induces the repression of Let-7, therefore leading to the overexpression of *MYC* [69].

Very recently, miR-22 has been identified as another miRNA involved in the regulation of *MYC* translation in MM cells [70]. In particular, a c-Myc/miR-22 feed-forward loop has been described in which c-Myc represses the transcription of miR-22 which in turn targets *MYC* mRNA. Interestingly, lenalidomide increases miR-22 expression in IMiD-sensitive patients leading to c-Myc downregulation. Accordingly, low miR-22 levels are associated with IMiD resistance in MM patients and miR-22 mimics restores drug sensitivity leading to synergistic anti-MM activity [70].

As to RNA binding proteins, Pourdehnad et al. demonstrated the association between mTOR-dependent 4EBP1 phosphorylation, *MYC* translation increment and MM cell survival. mTOR is a master regulator of protein synthesis control through direct phosphorylation of the tumor suppressor eukaryotic translation initiation factor 4E (eIF4E) binding protein 1 (4EBP1). This phosphorylation blocks the ability of 4EBP1 to inhibit the translation initiation factor eIF4E, thus promoting the recruitment of the 40S ribosomal subunit to the 5′-cap of mRNAs and enhancing translation initiation [71]. The role of mTOR-dependent 4EBP1 phosphorylation in c-Myc-driven myeloma was examined in the mouse model Vk*MYC. The authors isolated CD138^+^ plasma cells from Vk*MYC and wild-type mice and used a flow cytometry assay to directly evaluate and quantify 4EBP1 phosphorylation. Vk*MYC malignant plasma cells displayed increased 4EBP1 phosphorylation compared with wild-type plasma cells, thus suggesting that mTOR-dependent 4EBP1 phosphorylation is involved in promoting c-Myc translation from MM initiation to maintenance. Furthermore, the authors demonstrated that c-Myc-driven myelomas are druggable by a potent new class of mTOR active site inhibitors that are able to block 4EBP1 phosphorylation, suggesting that targeting mTOR-dependent phosphorylation of 4EBP1 may represent a therapeutic strategy to target c-Myc in MM [72].

### Mechanisms regulating c-Myc protein stability in MM

Finally, increased *MYC* signature activation in MM may also occur through different molecular events that alter c-Myc protein level by reducing c-Myc degradation and/or increasing c-Myc stabilization. Among these events, it can be counted the enhanced stabilization and accumulation of c-Myc protein caused by mutations in the RAS genes family [73]. RAS family mutations are present in about 20–30% of newly diagnosed MM and already found at lower rate in MGUS, suggesting their role in disease progression. Among the family members, NRAS and KRAS are the two most mutated genes in MM [74]. Importantly, RAS pathway activation stabilizes c-Myc through the phosphorylation of c-Myc at S62 residue mediated by the mitogen-activated protein kinase ERK, thus enhancing the half-life of the oncoprotein and preventing its proteolytic degradation [41]. Moreover, the Ras-dependent PI-3 K pathway is also critical to induce c-Myc protein accumulation through the inhibition of GSK-3β activity and thus the lowering of T58 phosphorylation. In this context, we have recently demonstrated that FGF/FGFR inhibition in MM can affect the activation of downstream RAS and PI-3 K pathways, induces GSK-3β activation and leads to c-Myc degradation, as discussed in the next paragraphs.

## The FGF/FGFR system

The system composed by the fibroblast growth factors (FGFs) and their receptors (FGFRs) is involved in several physiological processes, including angiogenesis, metabolism, tissue regeneration, embryogenesis, homeostasis and development [75]. The role of this system is mainly exerted by modulating migration, proliferation, differentiation, and apoptosis of target cells [76].

**Fig. 3:**
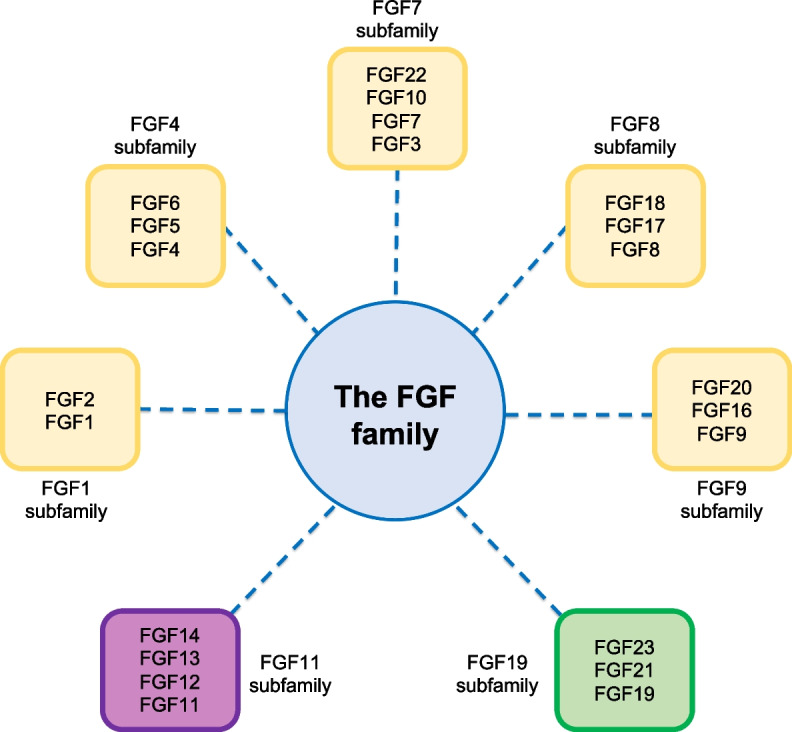
The FGF family. The FGF family is represented by 18 secreted members with paracrine/autocrine (canonical FGFs, in yellow) or endocrine (hormonal FGFs, in green) functions and 4 intracellular members (in violet)

The FGF family is represented by 18 secreted members with paracrine/autocrine (canonical FGFs) or endocrine (hormonal FGFs) functions and 4 intracellular members [77–80] (Fig. 3). The secreted members are divided into six subfamilies (FGF1, 4, 7, 8, 9 and 19) (Fig. 3), based on their sequence homology and phylogenetic analysis [81], and can interact with four tyrosine kinase (FGF) receptors named FGFR1, FGFR2, FGFR3 and FGFR4.

**Fig. 4:**
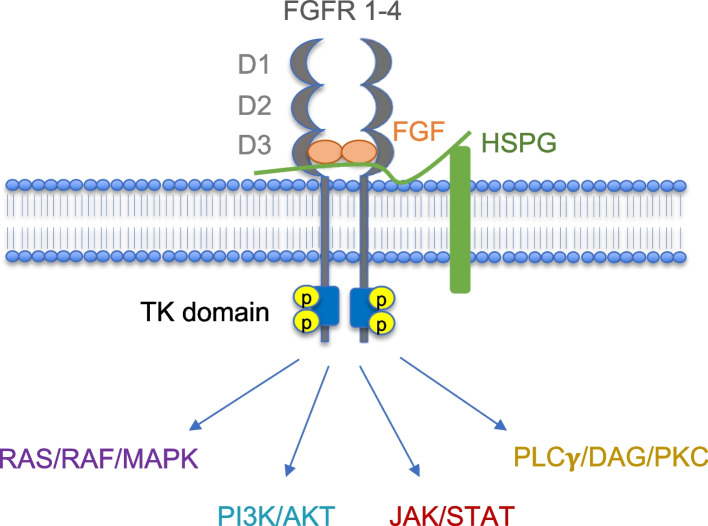
FGF signaling activation. FGFs binding to FGFR1-4 induces receptor dimerization and TK domain transphosphorylation. This in turn leads to the activation of downstream signaling pathways. HSPG, heparan sulfate proteoglycan. See text and Ref [76, 82, 84–86, 88] for further details

Structurally, each canonical FGFR is formed by three extracellular immunoglobulin-like domains (D1, D2 and D3), a single transmembrane domain and an intracellular tyrosine kinase (TK) domain (Fig. 4). The alternative splicing of D3 domain generates IIIb and IIIc isoforms of FGFR 1–3 receptors able to bind specific FGF ligands [82]. An additional FGF receptor, named FGFR-like 1 (FGFRL1) or FGFR5, has been described [83]. FGFR5 lacks the tyrosine kinase (TK) domain but maintains the ability to bind FGFs with high affinity, thus acting as a decoy receptor.

The FGF interaction with the four canonical FGFR leads to a series of receptor conformational changes culminating with the activation of intracellular signaling cascades (Fig. 4). The first step is the transphosphorylation of the tyrosine kinase (TK) domain that induces receptor dimerization and phosphorylation/activation of multiple downstream effectors (extensively reviewed in 77, 82, 84–87). The main intracellular signaling pathway starts with the phosphorylation of FGFR substrate 2 (FRS2) and phospholipase C-γ (PLC-γ) that in turn activate phosphoinositide 3-kinase (PI3K)/AKT and RAS/mitogen-activated protein kinase (MAPK). Additional transduction pathways can be activated, including STAT-dependent signaling, ribosomal S6 protein kinase and p38 MAPK (Fig. 4) [82, 86, 87].

An aberrant activation of the FGF/FGFR system often occurs in human cancers sustaining tumor cell proliferation and progression. In tumor cells, multiple genomic alterations including i) activating mutations [89], ii) gene overexpression/amplification [90], iii) chromosome translocation [91], and iv) aberrant autocrine/paracrine signaling and tumor-stroma crosstalk [92, 93] may affect various members of the FGF/FGFR family. These alterations may induce ligand -dependent or -independent FGFR activation and contribute to the dysregulation of this signaling pathway and its oncogenic functions in several cancer types, including MM.

In light of the pivotal role played by the FGF/FGFR system in tumor growth and progression, several therapeutic agents have been developed with different targeting, selectivity and specificity, including FGFR TK inhibitors, neutralizing monoclonal antibodies and FGF traps (extensively reviewed [85] and [94]).

### Impact of FGF/FGFR system deregulation in MM

**Fig. 5:**
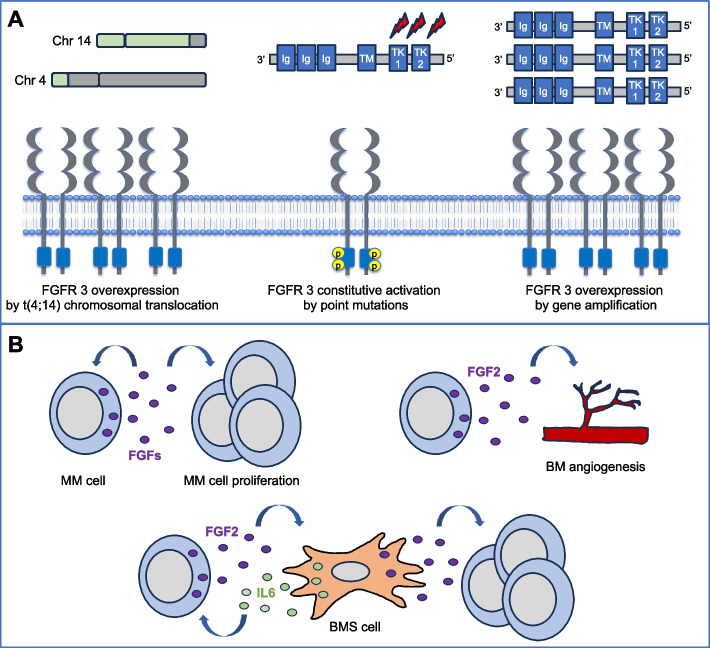
Deregulation of the FGF/FGFR system in MM.** A** Aberrant activation of the FGFR signaling can occur in MM as the consequence of genomic alterations affecting *FGFR3* gene, including t(4;14) chromosomal translocation, point mutations in the sequences codifying for the TK domain, or *FGFR3* gene amplification. **B** Aberrant activation of the FGFR signaling can also be due to increased production of FGF ligands by both MM and BMS cells. MM and BMS cells produce several members of the FGF family that stimulate MM cell proliferation and survival. FGF2 produced by MM cell can also stimulate BM angiogenesis and the secretion of IL-6 by BMSCs that in turn stimulate MM cell proliferation and FGF2 production

The crucial role of the FGF/FGFR signaling axis in MM is supported by the high level of expression of FGFRs in both MM cell lines and patient-derived MM and bone marrow stromal cells (BMSCs) [95–97]. For instance, the overexpression and aberrant activation of FGFR3 is oncogenic and associates with the initiation and progression of MM. In this context, almost 15% of newly diagnosed MM patients present the intergenic t(4;14)(p16;q32) translocation that involve both the *MMSET* and *FGFR3* genes [98]. The t(4;14) leads the *FGFR3* gene under the active control of the immunoglobulin heavy chain IgH enhancer, transcriptionally active in B cells, inducing a high and constitutive expression of FGFR3 [99, 100]. This strongly increases the FGF ligand-dependent FGFR phosphorylation causing an hyperactivation of the downstream pathways [96]. By contrast, *FGFR3*-activating mutations (e.g., A1157G, A761G, A1987G and G1138A) that lead to a ligand-independent receptor activation are observed in only a small percentage of t(4;14) MM patients [101, 102]. Also, rarely *FGFR3* gene amplifications are present in MM cell lines and MM patients causing an increasing number of copies of such gene [79, 103] (Fig. 5A). From a biological point of view, an aberrant activation of FGFR3 promotes the proliferation and survival of MM cells. Indeed, the t(4;14) translocation is clinically associated with poor prognosis due to a short progression-free survival (PFS) and aggressive relapses [104, 105]. Interestingly, recent data indicate that some patients lose the FGFR3 expression during MM progression, suggesting that therapies against FGFR3 could be more effective in the early stage of disease [106–108].

If the role of t(4;14) translocation is quite clear in full-blown MM, little is known about the role of aberrant FGFR activation in pre-malignant stages of MM. However, t(4;14) translocation has been found as early as MGUS, thus being known as a primary event in the development of MM [105, 109–111]. Consistently, t(4;14) translocation is suggested to confer a higher risk of progression from pre-malignant stages to MM because of (i) its enrichment in SMM, (ii) the higher prevalence in MM compared to SMM and MGUS, and (iii) the aggressive clinical course of MM characterized by t(4;14) [111, 112]. Despite this, some MGUS and SMM cases with t(4;14) translocation do not progress to MM [112] and do not present FGFR3 expression [113], suggesting more studies are needed to elucidate the role of FGFR aberration in earlier stages of MM.

An aberrant activation of FGFR signaling is observed also in MM cells not harboring the t(4;14) translocation [19], suggesting that other mechanisms beyond genomic alterations are involved in promoting the activation of this system, such as an aberrant production of FGF ligands by both MM and BMSCs (Fig. 5B). Indeed, BMS and MM cells secrete several FGFs in an autocrine/paracrine manner acting as potent proangiogenic factors and mitogenic cytokines [ 19, 95, 96, 114, 115, 116, 117]. In particular, high levels of FGF2, a potent pro-angiogenic protein, are involved in promoting the vascularization of BM that occurs during MM progression (Fig. 5B).

Accordingly, FGF2 is frequently detectable in both serum and BM of MM patients and associates with increase of disease activity [97, 118]. Importantly, myeloma cells seem to be the predominant source of FGF2 within the BM [97, 119], and FGF2 secretion plays a pivotal role not only in autocrine stimulation but also in mediating paracrine stimulation between tumor and stromal cells [95]. Indeed, it has been demonstrated that FGF2 may stimulate BMSCs to produce and increase interleukin-6 (IL-6) levels [120], a pleiotropic factor crucial for the growth and survival of MM. Importantly, FGF2-targeted therapies, such as anti-FGF2 antibodies, significantly hampered the secretion of IL-6 and anti-IL-6 antibodies inhibited the secretion of FGF2 by MM cells [95]. Thus, myeloma cells produce FGF2 that stimulates the production of IL-6 by BMSCs that in turn promotes FGF2 secretion by MM cells (Fig. 5B), finally creating a loop of stimulation between tumor and stromal cells [121].

Again, as for FGFR activation, scant data are available about FGF ligand expression during earlier stages of MM. Among all the members of the FGF family, FGF23 have been found enriched in the serum of MGUS patients. Importantly, FGF23 levels strongly correlate with the concentrations of markers of plasma cell burden such as serum paraprotein and β2-microglobulin. However, the absence of hypophosphatemia in these patients suggests that FGF23 produced by abnormal plasma cells is not systemically bio-active [122].

In keeping with the pivotal role exerted by the FGF/FGFR system in MM growth and progression, clinical trials are in progress to assess the effect of FGF signaling blockade by selective FGFR TK inhibitors in relapse/refractory MM patients (ClinicalTrials.gov). Accordingly, preclinical studies have demonstrated that both FGFR inhibition (by selective FGFR TK inhibitors) and extracellular FGF trapping (by the natural FGF trap PTX3 or the small molecule NSC12) are able to strongly reduce the proliferation and survival of MM cells co-cultured or not with BMS cells and to hamper MM cell proangiogenic potential [19, 117, 123]. Interestingly these studies highlighted not only the therapeutic potential of both FGF and FGFR inhibitors in MM, but also a strong link between FGF/FGFR signaling activation and c-Myc oncogenic functions in MM cells as discussed below.

## The FGF/FGFR/c-Myc axis in cancer

The presence of a link between the FGF/FGFR system and c-Myc, and its correlation with tumor establishment and progression has been suggested since 2010 when it was demonstrated that *FGFR3* cooperates with *MYC* in double transgenic (TG) mice to cause B lymphomas occurring with a higher penetrance and shorter latency than in single TG *MYC* mice [124, 125]. It is now well established that the oncogenic activity of FGFR is enhanced by co-expression of c-Myc in most cancer cells harbouring FGFR genetic aberrations [126]. Accordingly, tumor cells co-expressing c-Myc are more sensitive to FGFR inhibition [126] and c-Myc alteration can determine the therapeutic response to FGFR inhibitors [42], strongly suggesting that c-Myc is an important downstream mediator of the FGF/FGFR signaling.

**Fig. 6:**
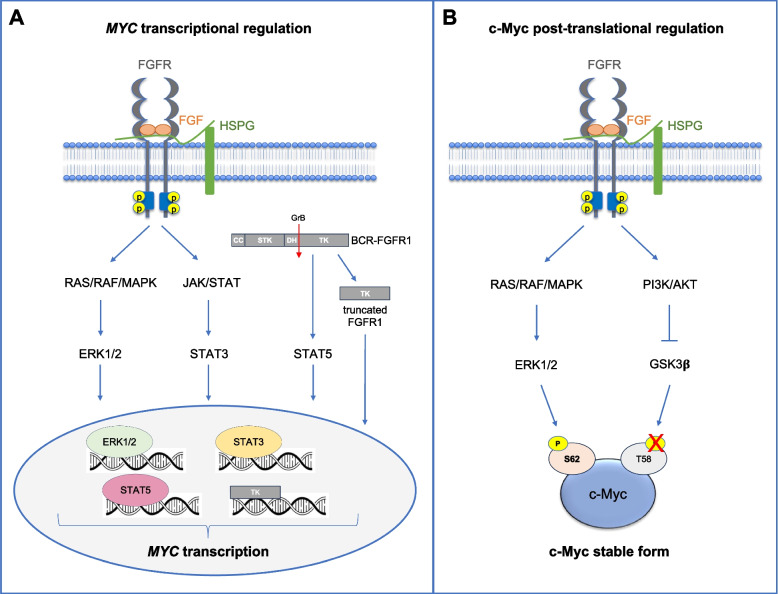
Regulation of c-Myc by the FGF/FGFR system in cancer. Activation of the FGFR signaling regulate c-Myc activity at both transcriptional and post-translational level. **A** FGFR phosphorylation activates transcription factors such as ERK1/2 and STAT3 that promote *MYC* transcription. Also, FGFR1 fusion kinase in the cytoplasm can activate STAT5 that contributes to *MYC* transcription. Alternatively, the truncated form of the FGFR1 fusion kinase generated from the cleavage by granzyme B can translocate to the nucleus where it recognises specific binding sites in the *MYC* locus having a direct effect on *MYC* expression. **B** FGFR phosphorylation activates the kinase activity of ERK1/2 that in turn leads to c-Myc S62 phosphorylation and c-Myc stabilization. At the same time, FGFR also triggers PI3K/AKT pathway that inhibits GSK-3β kinase, thus preventing c-Myc T58 phosphorylation and c-Myc proteasomal degradation

However, the mechanisms underlying the connection between FGF/FGFR signaling and c-Myc have been only recently investigated. Indeed, c-Myc modulation by the FGF/FGFR system occurs through a complex set of mechanisms which involves several signaling pathways and transcription factors leading to both transcriptional and post-translational regulation of the oncoprotein (Fig. 6).

### Transcriptional regulation of *MYC* by the FGF/FGFR signaling in cancer

Regarding *MYC* transcriptional regulation, the FGF/FGFR system can strongly influence *MYC* transcription in several types of cancer. Indeed, FGFR phosphorylation leads to the activation of transcription factors involved in *MYC* transcription, including STAT3 and ERK1/2 [79, 82, 127–130]. For instance, FGFR3 activation leads to *MYC* expression by enhancing the MAPK pathway in bladder cancer [131]. Also, beyond the classical activation of transmembrane FGFR, different cytoplasmic forms of FGFR1 chimeric fusion kinases have been correlated to *MYC* overexpression in stem cell leukaemia/lymphoma syndrome. In fact, it was demonstrated that FGFR1 fusion kinase in the cytoplasm can activate proteins such as the signal transducer and activator of transcription factor 5 (STAT5) that contributes to *MYC* transcription. Alternatively, the truncated form of the FGFR1 fusion kinase generated from the cleavage by granzyme B can translocate to the nucleus where it recognises specific binding sites in the *MYC* locus having a direct effect on *MYC* expression (Fig. 6A) [132]. In keeping with the capacity of the FGF/FGFR system to regulate *MYC* at the transcriptional level, FGFR blockade with the pan-FGFR inhibitor BGJ398 was proved to suppress *MYC* transcription in KKLS gastric cancer cell line [133].

Further supporting the existence of an FGF/FGFR/c-Myc axis, it is important to note that the FGF/FGFR system itself can be modulated at the transcriptional level by c-Myc both directly and indirectly. Indeed, c-Myc can directly induce FGFR2 and FGFR3 transcription in SNU-16 gastric cancer cell line [134] and in bladder cancer cell lines [131], respectively. Furthermore, Zhang et al. showed that c-Myc can indirectly trigger FGFR activation in pancreatic cancer cells by inducing the transcription of the fibroblast growth factor binding protein 1 (FGFBP1) which binds and releases immobilized FGFs from the extracellular matrix and chaperons FGFs to their receptors thus enhancing FGF/FGFR signaling cascade [135].

### Post-translational regulation of c-Myc protein by the FGF/FGFR signaling in cancer

Beyond *MYC* transcriptional regulation, the FGF/FGFR system is involved in c-Myc post-translational regulation as well, being correlated to c-Myc protein stabilization. Indeed, the levels of c-Myc protein rapidly decreased upon FGFR inhibition by the FGFR inhibitor PD173074 in FGFR-dependent H1581 and DMS114 human squamous cell lung cancer cell lines, leading to loss of the mitochondrial membrane potential and to cytochrome C release [126]. Also, c-Myc protein levels in H1581 and H520 cells strongly dropped down as early as three hours after treatment with the pan-FGF trap molecule NSC12 inducing oxidative stress and apoptosis [44]. Finally, treatment with NSC12 triggered the downregulation of the signal adaptor protein MYD88 in Waldenström Macroglobulinemia (WM), reducing c-Myc protein levels and *MYC* signature activity [136].

Based on this evidence, several studies have recently investigated the mechanisms by which the FGF/FGFR system can influence c-Myc stabilization (Fig. 6B). To this aim, as discussed above, it is important to remember that c-Myc protein stability is maintained by a strictly regulated system of phosphorylation according to which S62 phosphorylation by ERK_1/2_ or CDKs leads to protein stabilization and activation, whereas T58 phosphorylation by GSK-3β or BRD4 leads to ubiquitination and proteasomal degradation of the protein. In this context, the FGF/FGFR system seems to be able to control the phosphorylation status of both S62 and T58, leading to c-Myc stabilization with a synergistic double action. Indeed, in pancreatic cancer cells FGF1/FGFR1 signaling activates AKT pathway leading to GSK-3β phosphorylation and inhibition which finally results in decreased phosphorylation of c-Myc T58 and in reduced c-Myc proteasomal degradation. Concomitantly, c-Myc S62 phosphorylation was increased upon FGF1-mediated FGFR1 activation leading to c-Myc stabilization and increase of c-Myc levels and pancreatic cancer cell growth [137]. In MDA-MB-231 breast cancer cells Yu et al. showed that stimulation with FGF18 activates ERK pathway which boosts up c-Myc protein levels, thus favouring cell proliferation [138]. Moreover, aberrantly activated FGFR3 was reported to induce AKT pathway and GSK-3β inhibitory phosphorylation in bladder cancer cell lines, thus hampering c-Myc degradation [131]. According to these findings, further studies proved that inhibition of the FGF/FGFR system induces c-Myc protein degradation. Indeed, Ronca et al. showed that the inhibition of the FGF/FGFR system in MM cells by the pan-FGF trap molecule NSC12 and by the TK FGFR inhibitor BGJ398 results in the activation of GSK-3β kinase, thus triggering c-Myc ubiquitination and proteasomal degradation [19]. Also, Liu et al. demonstrated that BGJ398 and the pan-FGFR inhibitor AZD4547 can induce the blockade of the MAPK/ERK pathway in different kind of tumors, together with the downmodulation of AKT pathway which leads to increased phosphorylation of c-Myc T58 and eventually c-Myc degradation [42]. Finally, halting the FGF/FGFR system by the FGFR selective inhibitor ASP5878 was showed to reduce ERK phosphorylation and c-Myc protein levels in urothelial cancer cells [139].

Altogether these findings indicate that upon FGF binding to FGFR, activated FGFR induces several signaling pathways which regulate the c-Myc phosphorylation status. The activation of MAPK/ERK pathway leads to c-Myc S62 phosphorylation and c-Myc stabilization. At the same time, FGFR also triggers PI3K/AKT pathway which leads to inhibitory phosphorylation of GSK-3β, thus preventing c-Myc T58 phosphorylation and c-Myc proteasomal degradation (Fig. 6B). Finally, this double action (c-Myc stabilization and c-Myc decreased degradation) promoted by FGFR activation results in the increment of c-Myc protein levels with subsequent increased transcription of c-Myc target genes leading to tumor growth and progression.

These data are extremely important from the translational point of view of cancer treatment, since they strongly indicate the presence of an FGF/FGFR/c-Myc axis that could be efficiently targeted by FGF/FGFR inhibitors.

## Targeting the FGF/FGFR/c-Myc axis in MM cells

Being *MYC* deregulation one of the most relevant features among the genetic events that characterize MM, c-Myc targeting was believed to be a key in the pursuit of MM treatment. During the last decades, several strategies were developed to tackle MM by affecting *MYC*, but direct c-Myc inhibitors are still not yet employed in the clinic [73]. Indeed, the development of c-Myc inhibitors has been particularly difficult for the following reasons: (i) c-Myc protein resides in the nucleus, hence the need of molecules able to pass both through the plasma membrane and the nuclear membrane; (ii) the tertiary structure of c-Myc is intrinsically disordered, hence the impossibility of identify docking sites for candidate molecules; (iii) a large protein–protein interaction surface, which would not virtually benefit of small molecule inhibitors due to a spatial matter; (iv) the lack of a definite binding pocket, which is a consequence of (ii) and (iii); (v) the fact that the *MYC* family has 3 members, therefore the need to simultaneously inhibit c-Myc, N-Myc and L-Myc; (vi) being c-Myc ubiquitously expressed physiologically in a finely tuned fashion, its inhibition is believed to result detrimental for healthy cells and tissues [140]. Altogether, such evidence supports the reputation of c-Myc to be still “undruggable”. Indeed, the candidate inhibitors that were obtained so far showed low potency as well as poor pharmacokinetic properties, highlighting the need for a different approach to tackle c-Myc.

In the previous paragraphs we have reported the evidence that a strong connection exists between the FGF/FGFR system and c-Myc, being c-Myc activity strictly regulated by FGFR activation. This means that the FGF/FGFR/c-Myc axis could represent a promising therapeutic target in MM and that FGF/FGFR inhibitors could be exploited to indirectly target c-Myc oncogenic functions.

### Molecular effects induced in MM cells upon FGF/FGFR/c-Myc axis blockade

We have recently demonstrated that by inhibiting the FGF/FGFR system in MM, a complete blockade of c-Myc mediated signaling is achieved through induction of c-Myc protein degradation [19]. In this work, we first showed that an autocrine FGF/FGFR stimulation is essential for MM cell survival and progression. Accordingly, the blockade of the FGF/FGFR system in MM cells by either extracellular FGF trapping (by the small molecule NSC12), or FGFR inhibition (by the selective TK inhibitor BGJ398) hampered MM cell growth and dissemination both in vitro and in vivo. A subsequent deeper molecular analysis by gene expression profiling revealed that the activation of FGFR protects MM cells from oxidative stress-induced apoptosis. Indeed, FGF/FGFR inhibition induced mitochondrial oxidative stress, DNA damage and apoptotic cell death that were prevented by the antioxidant vitamin E or mitochondrial catalase overexpression. Interestingly, mitochondrial oxidative stress occurred as a consequence of glutathione depletion after FGF inhibition with the FGF trap NSC12. It is well known that intracellular glutathione levels are regulated by c-Myc activity [141]. Accordingly, gene set enrichment analysis on MM cells treated with NSC12 revealed a strong downmodulation of c-Myc targets, suggesting a reduction of c-Myc activity after FGF signaling inhibition. Indeed, FGF/FGFR blockade induced the rapid proteasomal degradation of c-Myc protein, as confirmed by pre-treatment of MM cells with the proteasome inhibitor MG132 that prevented c-Myc degradation upon FGF inhibition. Interestingly, the induction of c-Myc proteasomal degradation was mediated by GSK-3β kinase activation upon the inhibition of FGFR phosphorylation. Indeed, blockade of FGFR signaling hampered Akt activation, thus maintaining the kinase activity of GSK-3β that, as reported in the previous paragraphs, regulates the phosphorylation of c-Myc at T58. Importantly, the pivotal role of c-Myc in preserving MM cells from oxidative-stress mediated apoptosis was demonstrated by generating myeloma cells overexpressing an undegradable mutant form of c-Myc (T58A) [19]. Ectopic expression of this mutant form prevented c-Myc proteasomal degradation, GSH depletion, mitochondrial oxidative stress and eventually rescued cells from apoptosis induced by FGF/FGFR blockade [19].

In keeping with in vitro data, MM tumor xenografts grown in immunodeficient mice and treated with NSC12 showed a significant reduction of activated FGFR, increased activation of GSK-3β kinase and a strong reduction of c-Myc protein levels. This was paralleled by increased oxidative stress, DNA damage, inhibition of tumor cell proliferation and tumor vascularization, increased tumor cell apoptosis and finally delayed tumor growth compared with untreated mice. FGF trapping also hampered the homing of MM cell to BM niches and thus disease dissemination in zebrafish and mice models of systemic disease [19].

Importantly the presence of an FGF/FGFR/c-Myc axis was observed also in MM cells isolated from 26 newly diagnosed and relapsed/refractory MM patients. After treatment with NSC12, reduced FGFR phosphorylation and ERK 1/2, JAK2/STAT3 pathways were observed, together with downmodulation of c-Myc protein and increment of oxidative stress, DNA damage and apoptosis. Of note similar effects were observed both in newly diagnosed and relapsed/refractory patient-derived cells [19].

**Fig. 7:**
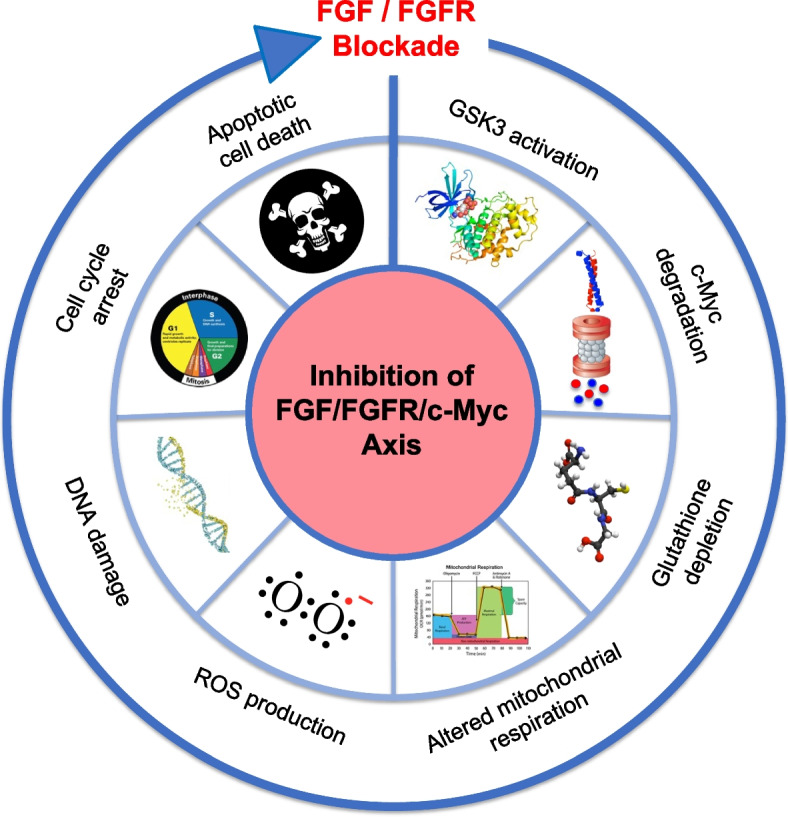
Effects of FGF/FGFR blockade in MM cells. FGF/FGFR inhibitors block the FGF/FGFR/c-Myc axis by activating GSK-3β kinase that rapidly induces the degradation of the oncoprotein c-Myc which in turn causes GSH depletion, mitochondrial oxidative stress, DNA damage, cell cycle arrest and eventually apoptosis of MM cells

Overall, these findings strongly prove the direct link between FGF/FGFR and c-Myc, identify the FGF/FGFR/c-Myc axis as a new therapeutic target for the treatment of MM and indicate that FGF/FGFR inhibitors can represent an efficient strategy to target the FGF/FGFR/c-Myc axis in MM cells (Fig. 7). Importantly, the disruption of FGF/FGFR/c-Myc axis may represent a valid therapeutic approach not only for newly diagnosed patients but also for those patients affected with relapsed/refractory MM.

## Conclusions and future perspectives

The present review article has provided an overview of recently explored connections between the FGF/FGFR system and c-Myc oncoprotein, sustaining the therapeutic potential of targeting the FGF/FGFR/c-Myc axis in MM by using FGF/FGFR inhibitors. Importantly, the provided findings may represent the rationale for using FDA approved FGFR TK inhibitors (i.e. Pemigatinib, Futibatinib, Erdafitinib) for the treatment of MM patients presenting with an aberrant activation of this axis. On these bases, it would be worthy to assess the levels of FGFR activation and c-Myc expression in patient-derived samples as a new predictive biomarker for the use of FGF/FGFR inhibitors in MM.

Finally, due to the potential implications of the small molecule NSC12 as an orally available FGF trap able to indirectly target c-Myc, novel NSC12 derivatives have been investigated in order to improve its chemical structure, potency and oral bioavailability [142, 143]. So far, two novel promising FGF traps molecules endowed with anti-myeloma activity have been identified [143] and could represent new potential FGF inhibitors available for further clinical investigation beyond the already approved FGFR TK inhibitors.

## Data Availability

No datasets were generated or analyzed during the current study.
